# Reported Motivations and Aims of Australian Dog Breeders—A Pilot Study

**DOI:** 10.3390/ani10122319

**Published:** 2020-12-07

**Authors:** Simone A. Blackman, Bethany J. Wilson, Alistair R. Reed, Paul D. McGreevy

**Affiliations:** 1 Tasmanian College of Business and Economics, University of Tasmania, Hobart, TAS 7005, Australia; 2 Sydney School of Veterinary Science, Faculty of Science, The University of Sydney, Sydney, NSW 2006, Australia; bethany.wilson@sydney.edu.au (B.J.W.); paul.mcgreevy@sydney.edu.au (P.D.M.); 3 Independent Researcher, Hobart, TAS 7170, Australia; areed@independentgeologist.com

**Keywords:** breeder, dog, breeding practices, hobby breeding, commercial breeding, dog attributes

## Abstract

**Simple Summary:**

In Australia, it is estimated that approximately 400,000 puppies are born each year. These are bred both by breeders affiliated with breeding associations and by others with no such affiliation. There is no way to measure accurately the number of people breeding dogs because there is no national requirement to register breeding activities and dogs are relatively easy to breed and sell without the keeping of records. The only accurate publicly available figures are the number of ANKC registered breeders of pure-breed dogs and the number of puppies that these breeders produce and register. In 2019, these breeders bred fewer than 67,000 puppies. Little is known about what motivates breeders and what breeding practices they adopt. Less still is known about whether breeders prioritize practices that ensure that puppies are suitable as companion animals. This article explores the reported breeding motivations, objectives, and breeding and selling practices of 275 breeders who undertook an online survey. It reveals that most respondents view their breeding as a hobby, and they breed because of a love for a certain breed of dog. They report that they seek to breed healthy companion animals and are committed to the long-term health of the puppies they are producing. When they decided to start breeding, most respondents recall that they were not motivated by money, but they currently report that the aim to make money on each litter is very important.

**Abstract:**

It is estimated that around 40% of Australian households currently own dogs that have been acquired from a variety of sources, including purpose-bred litters. However, little is known about how litters are being planned, whelped, and raised and less still on what motivates breeders to adopt their current practices. The current study used on online survey to explore the motivations and aims of Australian dog breeders; the breeding and selling practices Australian dog breeders favor and the extent to which breeders classify their breeding in terms of business, or hobby. Responses from breeders (n = 275) revealed that whilst most did not commence breeding to make financial gain, 86% of participants who answered the question confirmed that the making of money when they breed was a very important aim. Most breeders did not view their breeding as a commercial activity, despite nearly 20% of them confirming that they had declared income from the breeding and selling of puppies to the Australian Taxation Office.

## 1. Introduction

It is estimated that approximately 40% of Australian households currently own a dog and it is accepted that, for many Australians, owning a dog is a fundamental part of their lifestyle [1,2]. Prospective dog owners have a variety of sources available to them when considering acquiring a dog. They may choose to purchase directly from a breeder or from a third-party seller, be it a pet shop, rescue organisation, or shelter. All these dogs have been bred, either intentionally or unintentionally, by breeders with reasons or explanations for doing so. A large proportion of the resulting litters are born outside of the regulated environment, i.e., by breeders who are not in some way regulated by either industry or state or territory-based breeding codes and standards. It is not feasible for agencies tasked with enforcing breeding codes, standards, and selling regulations to reach into every home or breeding facility to monitor and log the breeding and rearing practices for every litter of puppies being born in Australia. Therefore, little is known about how litters are being planned, whelped, and raised and less still on what motivates breeders to adopt their current practices.

Before an examination of breeders’ motivations and objectives can occur, it is necessary to broadly categorise breeders. For the current study, we consider breeders within three groups: commercial breeders; pedigree hobby dog breeders; and occasional breeders. It is recognised that breeders might fall into one or two of these groups at any one time or shift from one to another over time. The first group are those breeders who breed to make money from having brood bitches produce puppies, standing dogs at stud, and from selling puppies. Some of these breeders run commercial facilities where dogs are kept in outside kennels. Their dogs are often treated as commercial breeding stock and not considered to be family members or companions. Some breeders who breed this way will comply with local dog welfare requirements such as the breeding codes that regulate dog breeding establishments in the Australian Capital Territory, New South Wales, and Victoria [3,4,5]. Others within this group may breed in ways that (often inadvertently) compromise dog welfare (e.g., those whom the public perceive to be ‘puppy farmers’).

The second group (for the purposes of the current article) are those breeders who breed pure-breed dogs (also known as pedigree dogs but referred to as pure-breed dogs in this article) as a hobby as a member of a state or territory canine association, such as Dogs NSW. As such, they are subject to property inspections when registering as breeders with that state breeding association [6]. They are also subject to state or territory industry codes of breeding ethics which direct that members shall breed only with the intent of maintaining and/or improving the standard of the breed (see below) and not specifically or predominantly for the pet or commercial market [7]. While some of these breeders will produce puppies on a scale that suggests a commercial element to their breeding, most breed fewer than four litters per year. For example, over 90% of the 32,543 Australian National Kennel Council Ltd. registered breeders who bred in 2016 bred fewer than four litters that year [8]. This group are the only breeders subject to rules that mandate they report on the number of puppies that they produce each year and this information is freely available as a resource collated by the Australian National Kennel Council, Ltd. [8].

The third group of breeders consisted in this article are the accidental and occasional breeders. They are often labelled ‘backyard breeders’, a pejorative reference to their relative lack of dedicated dog housing and breeding facilities. A large proportion of this group of breeders may breed outside of any established regulatory framework. Therefore, very little is known about why they breed and what breeding practices they adopt.

In the earliest days, dog breeding focused on breed utility and functionality. In contrast, these days most dogs are no longer bred for their original purpose [9,10]. Nevertheless, pure-breed dogs are judged against a standard that sets out the physical attributes that dogs of a given breed are required to exhibit. An Australian breed standard typically lists 18 or so attributes that a dog of a particular breed should possess to be considered a representative member of that breed; one from which litters can be bred [11]. Of these definitive attributes, only the one typically called ‘temperament’ refers to the dog’s behaviour and it goes largely untested in the show ring. The remaining attributes refer to morphological attributes that describe, for example, the morphology of the head and skull, body, feet, tail, movement or coat the dog should possess. To breed to the standard, breeders select dogs that they believe most closely align to the standard, as they interpret it.

Those dog lovers and advocates who do support dog breeding at any level usually advocate for breeding and rearing practices that enhance animal health and welfare. Integral to this is the adoption of breeding practices that ensure dogs make good companions and that reduce the chances of dogs being relinquished into shelters or pounds [10,12]. Veterinarians support breeding practices that promote dog health and welfare for both breeding dogs and puppies. Bennett and Perini believe that to do this, veterinarians must have no vested interest in maintaining breed standards or breed type [13]. However, those who advocate for the ongoing existence and breeding of pure-breed dogs have a vested interest in maintaining breed standards. Pure-breed dog breeders are compelled, by state and territory industry breeding codes, to breed with the object of maintaining and/or improving the standard of the breed and the health, welfare, and soundness of their dogs [6]. Breeders who are not regulated by either industry or state or territory breeding codes are not compelled to breed for any codified intent or objective. Nor are they compelled to report on how and why they breed.

Little is known about what motivates any breeder nor what breeding practices they engage in [12]. This is true irrespective of which of the three groups they fall into. It is important to all those who advocate for dog health and welfare to understand what motivates all breeders; those who breed under an industry code and those who do not. There is literature that suggests that many breeders do set health and welfare traits as priorities when they breed but there is little literature that examines this in detail [14]. Research that considers what priorities, motivations, and objective breeders aspire to will help to establish connections between breeder’s motivations, breeding practices, and the quality of puppies being produced and offered for sale.

Some breeders in any of our three groups may breed to make money but we acknowledge that financial gain may not be their only objective. The other objectives of breeders might be less straightforward as they may reflect a number of motivations for breeding. Breeders of pure-breed dogs may breed so that they can compete in conformation showing events and in other dog sports, such as agility, herding, obedience, and dancing with dogs [15]. The pursuit of these hobbies may be an objective, but so too may be the making of money to facilitate further breeding and participation in dog sports. Other breeders may breed to produce puppies for working roles, such as on farms, in law enforcement, and as guide dogs.

An awareness of these motivations will assist those who seek to understand which breeding objectives are the most likely to produce dogs that are behaviourally and physiologically suitable for certain roles, including the role of companion dogs [16]. Knowing what motivates breeders will enable the development and effective targeting of breeder education and support, as required. If breeders are motivated solely by profit, they would be expected to prioritise profit over and above welfare considerations. If, despite breeder education, desire for profit continues to vanquish concern for welfare, then perhaps more regulation and oversight are required. In contrast, if breeders are motivated by a love of dogs, then there may be merit in educational initiatives for both industry groups and other interested stakeholders. Such initiatives could focus on the merits of optimal dog welfare, irrespective of motives. Monitoring the motivations of breeders as trends in the popularity of breeds change [17] will also allow potential puppy purchasers to choose a puppy from a breeder whose breeding motivations and practices best align with their own values.

Of course, when asked, some breeders may be more transparent than others about the objectives and motivations that underpin their breeding. Irrespective of their primary motive, most breeders do not keep all the puppies they breed. Those that they do not keep are usually sold. Provided dog-seekers are aware of breeders’ motivations and breeding practices, it is possible that the more aligned a breeder’s objectives are to the production of healthy companion animals, the more dog-seekers will favour dogs bred by such breeders. Currently, it is difficult for dog-seekers to establish what motivates breeders. If industry or other stakeholder groups could provide information about the motivation of breeders, through initiatives such as breeder assurance schemes, potential puppy purchasers make more informed buying choices. This indicates a need to understand what breeding and selling practices are adopted by breeders and what breeders prioritise when they breed and sell their puppies. Alongside this is the reality that if breeders are aware that their breeding practices and motivations play a role in puppy-purchasing decisions, the more breeders might use breeding practices that promote the production of healthy companion animals. If buyers make this clear to puppy sellers by placing a premium on transparent breeding practices, such transparency should improve breeding practices.

The current study addresses three central research questions. First, what are the motivations and aims of Australian dog breeders? Second, what breeding and selling practices do Australian dog breeders use? Third, do breeders classify their breeding activity as a business or a hobby?

Knowing these motivations and aims will improve the public’s understanding of the dog-breeding landscape and assist in determining what changes, if any, by way of regulation, education and breeder support could ensure that the dogs of the future are bred in ways that allow breeders to retain a social license to operate [18].

## 2. Methods

### 2.1. Survey Design and Materials

Participants were Australian dog breeders who took part in an online survey. The questions were grouped into four sections about: objectives and motivations for breeding; selling and breeding puppies; the business of breeding; and membership of breeding organisations and demographics. A link to the full survey is provided in the supplementary material.

#### 2.1.1. Motivations and Aims of Breeding

Participants were asked four questions within this set of questions. Firstly, participants were asked to rank the importance of seven statements in the order that best described their motivation for breeding their first litter. They were asked to rank the following statements with 1 being the option that best described what motivated them to 7, being the least relevant motivation:

- *My love of dogs in general* [from here on labelled: Dog lover];
- *My love of a specific breed of dog* [Breed specific];
- *My love of particular dog I wanted to breed from* [Dog specific];
- *My love of competing in dog events* [Dog events];
- *Another breeder encouraged me to breed my first litter* [Breeder encouraged];
- *My family have been involved in dog breeding, so I continued the tradition* [Dog breeding family]; and
- *I believed that breeding would provide some financial benefit* [Financial gain].

Participants were also asked to rate the importance of 7 stated aims when they bred. Specifically, breeders were asked to score the following aims as: not important (0), somewhat important (1), very important (2) or essential (3):

- *To breed to the Australian National Kennel Council Ltd. (ANKC) Breed Standard* [Breed standard];
- *To breed fit and healthy companion animals* [Healthy companions];
- *For the betterment of the breed in Australia* [Breed betterment];
- *To breed dogs that are fit for their original purpose* (i.e., working or service dogs) [Original purpose);
- *To breed dogs that can win in dog events* [Dog events];
- *To make financial gain to allow me to continue to breed my dogs* [Finance breeding]; and
- *To make financial gain as a source of income* [Financial gain].

Breeders were asked to rank the importance of seven attributes of a stud dog and eight attributes of a brood bitch when selecting the parents for a planned litter. They were asked to assign 1 to the most important attribute and 7 or 8 to the least important attribute.

- *Conformation*
- *Health*
- *General (temper)ament* [Stud dog only]
- *(Temper)ament as a mother* [Bitch only]
- *(Temper)ament for showing*
- *(Temper)ament as a companion*
- *Ease of whelping* [Bitch only]
- *Longevity*
- *Colour and markings*

#### 2.1.2. Breeding Puppies

Participants were asked four questions about their selling and breeding practices to indicate their role was in the physical and mental health of puppies they sold. They were asked to consider the following four statements and to provide their level of agreement or disagreement with each, by selecting from a five-point scale of ‘strongly agree’, ‘agree’, ‘neither agree nor disagree’, ‘disagree’, or ‘strongly disagree’.

- *I take responsibility for the short-term physical health (up to 3 years of age) of the dogs I have sold.*
- *I take responsibility for the long-term physical health (beyond 3 years of age) of the dogs I have sold.*
- *I take responsibility for the short-term mental health (up to 3 years of age) of the dogs I have sold, and*
- *I take responsibility for the long-term mental health (beyond 3 years of age) of the dogs I have sold.*

Participants were also asked to indicate what health testing they undertook in prospective breeding stock. The options listed were: *elbow and hip dysplasia*; *progressive retinal atrophy*; *collie eye anomaly*; *cataracts*; *Legge Perthe’s disease*; and ‘*Other, please provide details’*.

#### 2.1.3. Business of Breeding

Participants were asked 12 questions about litters they bred in 2014. The year 2014 was selected because it was the last full year they had to breed before the survey was administered in 2015. Participants were asked how many litters were bred in 2014 and how many live puppies were born in those litters. They were asked how many brood bitches under 6 years of age they owned in 2014 and how often they believed a bitch of the breed that gave them their largest litter in 2014 should be bred from over her lifetime.

Participants were asked if they ever declared any money earned by them from breeding and selling puppies as income to the Australian Taxation office (ATO). They were also asked if their breeding program was recognised as a business by the ATO. Participants were asked to select from a list of three choices the term that best described their breeding. The three choices were: ‘*a hobby*’; ‘*a small business*’; or ‘*a commercial breeding enterprise*’. Participants were also able to select a fourth choice ‘*Other, please provide details*’.

#### 2.1.4. Membership of Breeding Associations and Demographics

Participants were asked five questions in this set. They were asked if they were a current financial member of any state or territory canine association and, if so, which one and for how long had they been a member. The survey ended with two questions relating to the participants’ age and location in Australia.

### 2.2. Procedure

This research was approved by the Human Research Ethics Committee (Tasmania) Network (approval no. H0013192). The survey was administered online and was anonymous. Positive consent was inferred from all respondents by participants reading the consent form at the beginning of the survey instrument and then continuing to provide responses to the questions.

### 2.3. Survey Distribution

The survey was open online for 6 weeks (from 3 July 2015 to 12 August 2015). It was constructed using commercial survey software (Survey Monkey, San Mateo, CA, USA). A web-link was created and distributed on several dog-related social media and Facebook groups. These groups were those that promoted discussion around dog ownership, dog walking, dog sport and dog breeding.

### 2.4. Response Rate

A total of 275 dog breeders completed the survey. As there are no figures on how many breeders operate in Australia, other than there being 32,481 registered ANKC breeders in 2015, the relative response rate could not be calculated [8].

### 2.5. Statistical Analysis

The data obtained from the questionnaire were downloaded into Excel spreadsheets and into R statistical and computing software for analysis [19]. The data set out in Section 3.1.1 below considers responses from all respondents, noting where necessary the number of participants that chose not to answer each question.

Because of the nature of the survey, it was not feasible to remove all incomplete cases. However, to ensure adequate engagement with the survey, the data that are set out in Section 3.1.2 considers a data set of participants who answered at least one question from the final section ‘Membership of a Breeding Association’ and who provided a complete record. This provided 151 complete records to consider. A further 4 respondents were removed because they had more than 20 brood bitches. The decision to exclude these breeders was in combination of the small size of this sample group and in consideration of regulation that operates in Australia that confirms that breeders with this number of fertile female dogs must be approved to operate as a commercial dog breeder [20]. A hierarchical cluster analysis was undertaken on these 147 remaining breeders. The distance method (daisy algorithm from cluster analysis) and a Ward’s clustering agglomeration method were used.

## 3. Results

### 3.1. Motivations, Aims, and Attributes to Look for in Breeding

#### 3.1.1. Motivations, Aims, and Attributes to Look for in Breeding (n = 275)

Table 1 shows the respondents’ ranking of motives for breeding their first litter. It also includes the median and mode ranking for each of the motives, as nominated by survey participants. Table 1 reveals that over half of all the breeders who participated in the survey ranked their love of a specific breed as the primary motive for breeding their first litter. It must be noted that between 10% and 20% of respondents, depending on which statement they were ranking, chose not to provide an answer to this question in the survey. Of those who did, close to 84% ranked their love of a specific breed among their top three reasons for breeding. This motive ranked number 1 by mode and by median irrespective of adjusting by replacing or not replacing the blank responses.

The motive that ranked the lowest was the belief that breeding would provide some financial benefit. Only 5% of all respondents who answered this question ranked it in their top three reasons for breeding their first litter. Table 2 shows the results of the ranking of seven stated aims for breeding currently (i.e., not with reference to their first litter).

Of the seven motives offered to survey participants, the breeding of healthy companions was ranked first, by a total of 791 and 91.27% of participants, confirming that this aim was essential when they breed. Ranked second was breed betterment, with 78.55% of participants confirming that this was an essential breeding aim. The lowest ranked aim was breeding to make enough financial gain to continue breeding, with only 1.45% of participants stating that this was an essential aim in breeding and 58.18% stating it was not important. The aim of breeding for financial gain was ranked fifth among the seven motives offered. While only 0.73% of breeders confirmed that aim of breeding for financial gain was essential, 84.73% confirmed it was a very important breeding aim for them. Table 3 shows the results of the ranking of seven candidate attributes for stud dogs and Table 4 shows the ranking of eight attributes for brood bitches.

Both tables show that the attribute considered the most important when selecting breeding dogs is the focal dog’s health. For males, 68.36% of breeders ranked stud dog health as their first or second most important attribute. If we exclude the 25 respondents who selected ‘no response’ (and thus reduce to 250 the total number of respondents who answered), 75.20% of those breeders ranked stud dog health as their first or second most important attribute. Considering instead the health of bitch, health was even more important, with 73.82% of breeders ranking it first or second. After excluding the 25 non-respondents, we found that 81.2% of all breeders who rank bitch health as their first or second most important attribute when selecting a bitch to breed. The least important selection attribute for both stud dog and bitch was colour and markings, with only 7 participants indicating it was their first or second most important attribute (2.55% for both dog and bitch or, if we exclude the ‘no response’ participants, 2.82% and 2.91% respectively). Conversely, 67.64% and 58.91% said that a dog or bitch’s colour and markings were their least or second least important attribute when selecting a dog and bitch to use in breeding.

The second most important attribute for a stud male was its general temperament, with 44.73% of participants who responded to this question ranking this attribute as first or second in priority. Close third was confirmation, with 39.64% ranking this as the first or second most important attribute for a stud male. For bitches, conformation was the second most important attribute with 40.36%, of those who answered, ranking this as first or second most important attribute, followed by temperament as a companion, with 34.18% ranking this as first or second.

#### 3.1.2. Motivations, Aims, and Attributes to Look for in Breeding (n = 147)

**Figure 1:**
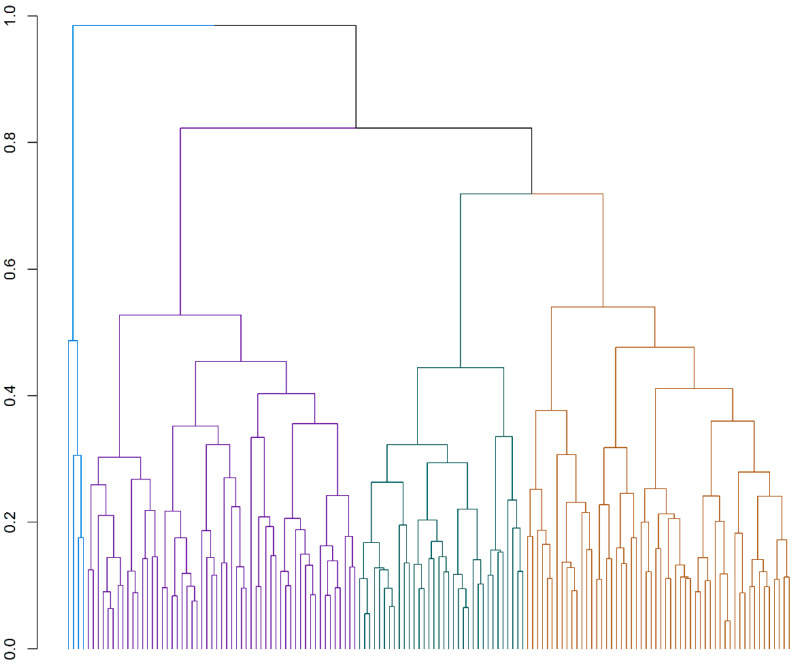
Cluster dendrogram and cluster analysis of motivations and aims for registered breeders (n = 147).

The motivations and stated aims of registered breeder respondents were clustered using Ward’s analysis to reveal four clusters of breeders (see Figure 1).

**Figure 2:**
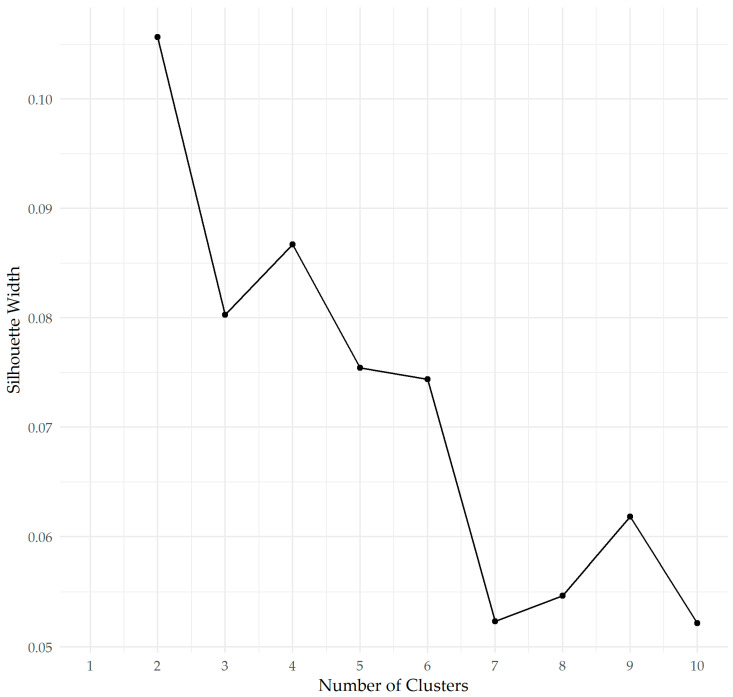
Elbow plot for clusters of registered breeders (n = 147) who responded to the current survey.

The clustering was checked against an elbow plot using Metroid clustering (PAM algorithm from cluster package [19], again using the Gower distance from the daisy algorithm; see Figure 2).

Figure 1 confirms two or four clusters. We have labelled them Purple (n = 55 respondents), Brown (n = 54), Green (n = 34), and Blue (n = 4). Table 5 provides an ordinal score ranking for the 147 registered breeders who provided a complete data set. The ordinal score is obtained by attributing a score to each ranking. A score of 7 is attributed to a ranking of most important, all the way down to 1 for the motivation that the breeder scored as the least important.

The Green cluster and the Purple cluster rank the love of a breed as their highest motivation. Those in the Brown are not far behind, with an ordinal score of 6.5. The remaining motivators were assigned middle ranking by the Green, Brown, and Purple clusters. The Blue group stands alone as a small cluster but is differentiated by indicating that dog’s breeds and love of events are all very low-level motivators. The Blue cluster was strongly polarised from all other groups by indicating a primary financial motivation for breeding. The Blue clusters financial gain motivation scores were significantly higher (coefficient = 5.205, t = 13.8, *p* < 0.001) than the Purple cluster whereas the Brown (coefficient = −0.120, t = −0.857, *p* = 0.393) and Green (coefficient = 0.043, t = 0.269, *p* = 0.788) clusters scores were not lower.

Table 6 reveals that the breeding of healthy companions and breed betterment were reported as being essential breeding aims across all clusters. All clusters indicated that breeding for financial gain was not important, but the Blue cluster indicated that finance to fund their breeding was somewhat important. The need to breed champions was more important to respondents in the Brown and Green clusters than it was to the Purple and Blue clusters. These data reveal that only the breeders in the Blue group report including some financial considerations in their breeding plans, i.e., aim to make some money to finance breeding. The other three clusters do not place importance on financial gain and instead aim to breed irrespective of the cost.

Level of importance of the breed standard was significantly higher in the Brown (coefficient = 0.580, t = 4.92, *p* < 0.00) and Green (coefficient = 0.5434, t = 4.05, <0.001) clusters compared to the Purple cluster, and significantly lower in the Blue cluster (coefficient = −1.060, t = −3.325, *p* = 0.001).

The data reveal that breeders have several aims when they breed. All breeders aim to breed fit and healthy dogs and to improve the breed. No cluster indicated that source of income was an essential or even a very important aim of their breeding. All clusters indicated that being able to attend dog events was either very or somewhat important. However, this aligns poorly with the responses from the Blue cluster when asked about what motivated them to start breeding. Table 5 shows that their primary motivation to start breeding was to make financial gain. This would suggest that they were motivated by money when breeding their first litter but that their aims once they had become a breeder changed to prioritise the breeding of healthy companions and breed betterment. Table 7 provides cluster medians for importance of sire attributes.

When ranking attributes or characteristics of potential stud dogs that dogs’ health ranked only slightly higher to or equal to the animal conforming to a breed standard for all clusters apart from Blue. For the Blue group, the dog’s conformance to the breed standard was more important than its health. None of the four clusters stated anticipated longevity as important. This may be because the time that the participant breeders had been breeding will vary and some may not have experienced the importance of longevity as a factor in their breeding activities. The Blue cluster, who stated that their main motivation to commence breeding was to make money, also prioritise colour and marking over temperament of their sires. Table 8 provides cluster medians for importance of dam attributes.

The health of the bitch is of utmost importance to the Purple and Brown clusters, just under that for the Green cluster (coefficient = 0.171, t = 0.72, *p* = 0.471) and not that important for the Blue cluster (coefficient = −4.682, t = −8.33, *p* < 0.001) who instead place the most importance on colour and marking and temperament for showing. To the contrary for clusters 1 to 3, colour and marking is almost irrelevant. The Blue cluster who were motivated primarily by the making of money to start breeding see the bitch’s colour as critical. Given that some combinations of coat colouring are related to health issues such as blindness and deafness [21] this focus on a breeding animal’s colour to produce puppies of certain colours is of concern.

For the Purple cluster, the second most important attribute is the bitch’s temperament as a companion, second only behind her health. This is in great contrast to the importance of her temperament as a show bitch. Maternal temperament is not at all important to the Blue group but important for clusters 1–3. Show temperament is not at all ranked highly for the Purple cluster and the Brown cluster but it very important to the Blue cluster. The Blue cluster is less concerned about conformation with a standard than the Purple and Brown clusters. The importance of temperament as a companion varies widely between groups. The Blue cluster attributes little importance to temperament in contrast to the Purple group.

In summary, for the Blue cluster, the attributes valued in both stud dog and breeding bitch differ from the other three clusters. The Blue cluster prioritise colour and markings over all other attributes. Health is the most important attribute for the Purple and Brown clusters and the second most important one for the Green cluster behind conformation.

### 3.2. Breeding Practices

Breeders were asked a set of questions to establish the extent of their breeding practice and the practices that they use in their breeding.

#### 3.2.1. Scale of Breeding

Breeders were asked how many litters they had in 2014. Of the 234 who answered this question, 32% confirmed they did not have a litter that year. Of those who had a litter that year, 34% confirmed they had one litter, 17% had two litters, 7% had three litters, 3% had four litters, 3% had five litters and 1% had six litters. Of the remaining 7 breeders, 2 had 8 litters, 1 had 13 litters, 1 had 19 litters, 1 had 20 litters, and 1 had over 25 litters. Breeders were then asked, if they bred in 2014, how many live puppies were produced.

**Figure 3:**
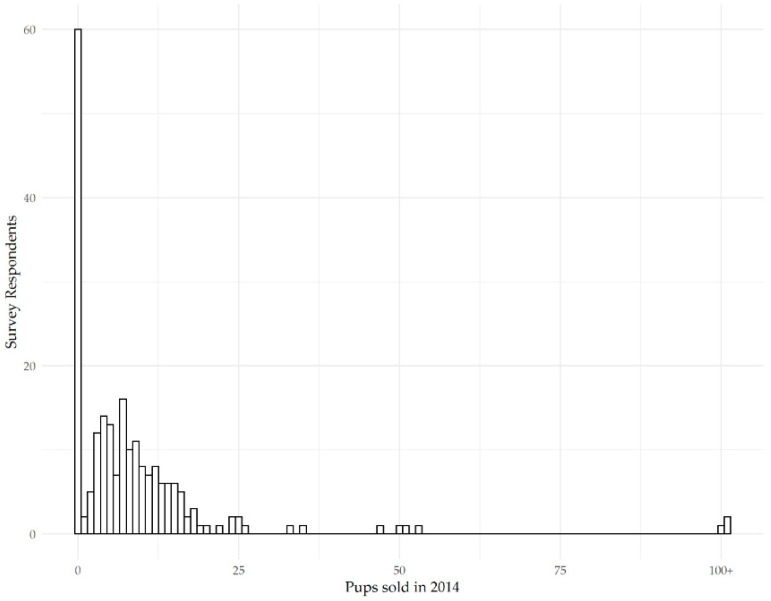
Puppies born to respondent breeders (n = 234) in 2014.

Figure 3 shows that of the 159 breeders who did have litters in 2014, 27.7% of them had five or fewer puppies, 32% had between 6 and 10 puppies, 20% had between 11 and 15 puppies, 7% had between 16 and 20 puppies, 2.5% had between 21 and 25 puppies, 3% had between 26 and 50 puppies, 2.5% had between 51 and 100 puppies, and 1.2% had over 100 puppies.

When participants were asked how many brood bitches they owned, 56 participants chose not to answer this question. Of the remaining 219 participants, the vast majority 86.75% owned 4 or fewer brood bitches that were under 6 years of age. Of those remaining 22 (10%), participants had between 5 and 10 brood bitches of breeding age and only 7 (3%) had over 10 brood bitches under 6 years of age.

#### 3.2.2. Health Testing

Participants were asked if they undertook health testing on their prospective breeding stock. The most common testing reported was for elbow and hip dysplasia, with over 57% of participants confirming that they undertook this type of testing. Only 8% of participants confirmed that they undertook no health testing on prospective breeding stock. The main finding was that 92% of participants reported some sort of health testing of their breeding stock prior to breeding.

#### 3.2.3. Taking Responsibility for the Health of the Puppies that They Produce

Participants were asked about the responsibility that they took for the health of the puppies that they bred. The results are set out in Table 9.

When asked if they took responsibility for the physical and mental health of the puppies that they produce, approximately 14% of participants elected not to respond. Of those who chose to answer these questions (n = 237), 63% either strongly agreed or agreed that they took responsibility for the short-term physical health of the puppy crop whereas 45% strongly agreed or agreed that they took responsibility for the long-term physical health. When it came to the mental health of puppies, 51% of participants strongly agreed or agreed that they took responsibility for the short-term mental health of the puppies that they produced whereas 34% strongly agreed or agreed they took responsibility for the long-term mental health of their puppies.

#### 3.2.4. Retention of Puppies

Breeders were asked how often they retained a female and male pick pup from the litters that they bred. Table 10 shows the results.

This reveals that breeders retain a pick female puppy more often than a male puppy, with over 54% of breeders always or often retaining a pick female puppy and only 23.34% of breeders retaining a pick male puppy (Wilcoxon ranked sign test: V = 8959, *p*-value < 0.001). When it comes to not retaining a pick puppy, just under 3% of breeders never retained a female puppy but 6.67% never retained a male puppy.

### 3.3. Business of Dog Breeding

Of the 251 participants who answered the question about how they would describe their breeding activity, 189 (68.73%) described their breeding as a hobby. After excluding the 49 non-respondents, this percentage increased to 85.52%. 23 participants described their breeding under ‘other’. Of the 23 respondents who selected ‘other’, 8 described their breeding as a hobby and/or a showing activity but they sought to expand or explain what hobby or showing meant to them. This increased the total who described their breeding as a hobby to 87.17% of participants who responded to the question. Just 1.82% described their breeding as a commercial breeding enterprise while 9 breeders (3.27%) described their breeding as a small business.

Participants were asked if they had ever declared any money that they had earned from their breeding to the Australian Taxation Office. They were also asked if their breeding was recognised as a business by the Australian Taxation Office. 21.4% of breeders chose not to answer this question. Just under 15% (41 breeders) confirmed that they had declared earnings from breeding to the ATO as income and 63.7% confirmed that they had not. Of the breeders who stated that they had declared income, just 12% of them (26 breeders) confirmed that their breeding was recognised as a business by the ATO.

## 4. Discussion

This article broadly surveyed the Australian dog breeding community expecting to gather data on commercial breeders, pedigree hobby dog breeders and occasional breeders. However, the current respondents were predominantly small pedigree hobby breeders, so our report focuses on the motivations and aims for such breeders.

### 4.1. Motivations, Aims, and Attributes to Look for in Breeding

The veterinary literature confirms that it is important to understand what motivates breeders. For example, Collins et al. [14] considered the question of breeding objectives and confirmed the putative benefits of appropriate breeding objectives in reducing the prevalence of inherited disorders in pedigree dogs. They suggested that the process of improving the quality of life for pedigree dogs starts with the development of effective breeding objectives with an overall goal of the breeding of healthy, long-lived companionable pedigree dogs. The current study reveals some of the breeding objectives of Australian dog breeders by reporting their motivations, aims and the attributes that they prioritise in their breeding dogs.

Several factors motivated respondent breeders to produce their first litter but the predominant factor was the love of a certain breed, with more than 73% of respondents nominating this as either their first or second most motivating factor when deciding to breed. Most respondents were not motivated by financial gain when they decided to commence breeding. It is only after breeding their first litter, that breeders can consider if they will continue to breed and what they hope to achieve with their breeding. Over 91% of participants indicated that the breeding of healthy companions was an essential breeding aim and a further 6.5% confirmed it was a very important aim. The lowest ranked aim was breeding to make enough financial gain to continue breeding, with 58.18% of participants stating it was not important and only 1.45% nominating this as an essential aim in breeding. While only 0.73% of breeders confirmed that the aim of breeding for financial gain was essential, 84.73% confirmed it was a very important breeding aim for them. This suggests that breeders do recognise that they can make financial gain when they breed but that profit is not their primary reason for breeding. Clearly, financial gain can cover some costs associated with attending shows or transporting bitches to studs.

When participants were asked to indicate the importance of a number of attributes in their breeding stock, 47% ranked the health of the stud dog as their most important priority. For breeding females, 52% ranked the health of the mother as their number one ranked priority. Only 1% ranked anticipated longevity of the male as their first priority. Indeed, only colour and marking and temperament for showing were assigned fewer number one rankings. This finding supports the view that most breeders focus on the attributes of young animals when they select breeding stock and that breeding for longevity is rare, even though dog owners value this trait [22].

An interesting finding is that, when breeders were asked to rank the importance of temperament as a companion, 10% ranked it as their number one priority when choosing a stud dog whereas 21% ranked it as their number one priority when selecting a breeding bitch. This aligns with the current finding that twice as many breeders prefer to retain females than males. One possible explanation for this preference is that breeders are more inclined to retain female dogs than males (and so are more concerned about temperament) because they have to live with the consequences of their choice (of females), but do not have to live with the stud dogs. There may, of course, be other explanations. These might be explored should a larger population be sampled in the light of the current pilot study.

Arguably, more meaningful results emerged when a cluster analysis was undertaken of the 147 breeders who provided a complete response to all questions in the survey and who confirmed they were members of a state or territory breeding association. Specifically, this revealed differences in the importance in motivations, breeding aims and the attributes that these breeders prioritise when breeding. This cluster analysis revealed four clusters that this study has labelled the Purple cluster, the Brown cluster, the Green cluster and the Blue cluster. The biggest difference among these clusters was seen in the Blue cluster of four breeders who disclosed that they were motivated to breed for financial gain and who prioritised colour, marking and breeding to the breed standard over the breeding of healthy companionable pedigree dogs. This would suggest that this group are more concerned with how a dog looks than the viability of its offspring as companions. That they prioritise the temperament of a dog for showing over its temperament as a companion suggests that they are concerned more with how these dogs look and perform while being shown than the dog’s temperament as a companion. These four breeders comprised a breeder of Dachshunds, two Rhodesian Ridgeback breeders, and one breeder who identified as having bred six different breeds during their time as a breeder. All four of these breeders had fewer than five brood bitches and all bred fewer than five litters in 2014. All self-identified as hobby breeders yet confirmed that they started breeding to attempt to make financial gain. Given the small number of breeders in this cluster, it would be unwise to dwell on these findings, but they may warrant more research beyond this pilot study.

Turning to the other three clusters, the Purple cluster, which comprised 55 breeders, seemed to be less motivated by conformation showing (love of dog events) than the Brown or Green clusters in that love of dog events was reportedly less relevant to them than breeding to the breed standard. The Purple cluster also ranked the breeding of champions as less important than the Brown and Green clusters did. This was despite their assigning a similar level of importance to the love of a specific dog breed as the Green and Brown clusters. The Purple cluster also assigned more importance to selecting breeding animals with a good temperament for companionship than the Green or Brown clusters. It is possible that the behavioural attitudes of successful show dogs, including boldness (that maybe labelled showmanship), partially align with some of those valued by potential puppy purchasers seeking a companion animal [23,24].

The Brown and Green clusters, that contained 54 and 34 breeders respectively, reported median motivations for breeding and breeding aims when producing a litter that were quite similar to one another. Differences between these two clusters emerged when one compared the importance assigned to various attributes of breeding dogs. Brown cluster breeders assigned more median importance to health, longevity, and whelping ease, whereas the Green cluster were more concerned with their breeding dogs’ show conformation, and their temperament as show dogs and companions.

In summary, all clusters except Blue were motivated by breeding a specific breed and not by finance (profit). Conversely, the Blue cluster was apparently motivated primarily by financial reasons; with no strongly stated love for a specific breed, dogs in general or love of showing. It is recognised that litter size varies considerably across breeds [25]. Avenues for further research in this domain could include establishing the role, if any, of breed-related fecundity in more detailed commercial characterisation of the clusters we have revealed. With a larger sample size, the scale of risk of health problems within each cluster also merits further analysis.

### 4.2. Breeding Practices

This study reports chiefly on respondents who are small breeders, with close to 93% of participants breeding between 0 and 4 litters in 2014 and 87% confirming that they owned four brood bitches or fewer. Only 7% of respondents bred more than 5 litters in 2014, only 10% owned between 5 and 10 brood bitches and only 3% owned over 10 brood bitches under 6 years of age in 2014. The current data revealed that only 57% of participants had a litter in 2014. Of these, close to 80% confirmed that they produced fewer than 15 puppies per year and 17% had fewer than 100 puppies per year. Only 1.2% of breeders reported producing more than 100 puppies in 2014.

When respondents were asked if they took responsibility for the physical and mental health of their puppies, more than half confirmed that it was their view that they had responsibility for both the short-term physical and mental health of the puppies that they produced. These percentages declined when respondents were asked about the longer-term health of puppies, but 45% still acknowledged some responsibility for the physical health of their puppies even beyond 3 years of age and 34% took responsibility for the mental health of the puppies beyond this age. This suggests that more than one third of all breeders had concern for their puppies into the longer term, but that physical health is more important to them than mental health. Given that, in the UK at least, behavioural disorders are the biggest cause of euthanasia in dogs under three years of age [26], it would be helpful to explore ways in which this imbalance of priorities could be redressed.

With respect to health testing, as each breed in Australia has its own set of genetic health issues—only some of which can be tested for—there were no data collected for this report that would allow consideration of the appropriateness or the quality of the testing being undertaken.

### 4.3. Business of Dog Breeding

Over 87% of respondents described their breeding as a hobby. Just under 2% (n = 5) described their breeding as a commercial breeding enterprise and just over 3% (n = 5) described their breeding as a small business. This is an interesting finding and suggests that most breeders of pedigree dogs do not regard breeding as a commercial activity. However, when asked if they had ever declared money from breeding to the Australian Taxation Office, 19% of breeders who answered the question (n = 41) confirmed that they had. Of these breeders, over 63% confirmed that their breeding was recognised as a business by the Australian Taxation Office.

Given that some puppies in Australia can be sold for several thousand dollars, the finding that close to 20% of all respondent breeders declared income is not surprising. However, what is notable that, when asked the simple question about how they view their breeding, less than 5% confirmed they viewed it as either a commercial breeding enterprise or small business. This apparent mismatch warrants further investigation in future.

### 4.4. Limitations

This pilot study is based on the results of data produced from a survey of 275 participant breeders who voluntarily undertook a survey. We accept that beyond the small sample size, there are a number of limitations and we urge the use of caution in interpretation of the results. As with any online survey, we cannot be sure that the respondents are truly representative of the general population. The results rely on the accuracy of the recollections and honesty of the participants. Although the aims and motivations were scored as ranks, they were converted to ordinal scores. This conversion assumes an equal distance, or score separates ranked criteria. This may have lost some subtleties in the data and acknowledge this as one of the current shortfalls from a notional ideal quantitatively analysis. For example, a breeder might rank attending events over devotion to a specific breed, but, in ordinal terms, the difference between ranked items might not be the same between each element in a list of options.

We acknowledge that the data come from responses made in 2015 and that they must be regarded as historic. Furthermore, we acknowledge that some of the terms in the current questionnaire may have been open to interpretation. For example, in asking whether breeders took responsibility for various aspect of their puppies’ future, we may have overlooked the prospect that some respondent may have interpreted this as total responsibility, whereas others may have interpreted it as partial responsibility. Other possible sources of bias with online surveys of animal owners, including the prospect that participants may sanitise their responses have been discussed elsewhere [27].

## 5. Conclusions

This pilot study surveyed Australian dog breeders to examine their motivations and the aims of their breeding practices. As a scoping exercise, it revealed a great diversity among the priorities respondents reported when selecting dogs for breeding, retaining home-bred pups, and selecting temperament for companionship.

## Figures and Tables

**Table 1:** Numbered ranking (and percentage distribution) of all 275 breeder respondents’ historic motives for breeding their first litter.

Breeding Motive							
	Dog Lover	Breed Specific	Dog Specific	Dog Events	Breeder Encouraged	Dog Breeding Family	Financial Gain
	n (%)	n (%)	n (%)	n (%)	n (%)	n (%)	n (%)
Ranking							
1	23 (8.36)	144 (52.36)	27 (9.82)	19 (6.91)	26 (9.45)	16 (5.82)	4 (1.76)
2	45 (16.36)	56 (20.86)	53 (19.27)	37 (13.45)	30 (10.91)	14 (5.09)	5 (2.20)
3	51 (18.55)	29 (10.55)	63 (22.91)	53 (19.27)	32 (11.64)	9 (3.27)	3 (1.09)
4	73 (26.55)	7 (2.55)	48 (17.45)	48 (17.45)	39 (14.18)	6 (2.18)	4 (1.76)
5	40 (14.55)	2 (0.73)	35 (12.73)	53 (19.27)	62 (22.55)	18 (8.22)	12 (4.36)
6	7 (2.55)	3 (1.09)	9 (3.27)	20 (7.27)	32 (11.64)	80 (29.09)	68 (24.73)
7	3 (1.09)	6(2.18)	2 (0.73)	12 (4.36)	9 (3.27)	76 (27.64)	131 (47.64)
No answer	33 (12.00)	28 (10.18)	38 (13.82)	33 (12.00)	45 (16.36)	56 (20.36)	48 (17.45)
Total	275	275	275	275	275	275	275
Median (leave blanks)	4	1	3	4	4	6	7
Mode (leave blanks)	4	1	3	4	5	6	7
Median (replace blanks)	3	1	3	3	4	6	6
Mode (replace blanks)	4	1	3	3	5	6	7

**Table 2:** Distribution and rating of importance of respondents’ (n = 275) current aims for breeding. [DOI] = Degree of importance is a summed total across the results for all participants who rated an aim as essential (3), very important (2), and somewhat important (1). Both not important and no response added 0 to the score. Each of the aims received a grand total on this basis and a ranking.

	Breeding Aims						
Rating	Breed Standard	Healthy Companions	Breed Betterment	Original Purpose	Dog Events	Finance Breeding	Financial Gain
	n (%) [DOI]	n (%) [DOI]	n (%) [DOI]	n (%) [DOI]	n (%) [DOI]	n (%)	n (%)
Essential (3)	190 (69.09) [570]	252 (91.27)	216 (78.55)	181(65.82)	37(13.45)	4 (1.45)	2 (0.73)
Very important (2)	53 (19.27) [106]	18 (6.55)	35 (12.57)	63 (22.91)	104(37.82)	16 (5.82)	233 (84.73)
Somewhat important (1)	13 (4.73) [13]	2 (0.73)	13 (4.73)	19 (6.91)	96(34.91)	89 (32.36)	27 (9.82)
Not important (0)	12 (4.36) [0]	1 (0.36)	6 (2.18)	8 (2.91)	32(11.64)	160 (58.18)	5 (1.82)
No response (0)	7 (2.55)	3	5	4	6	6	8 (2.91)
Grand Total of DOI	689	791	731	688	415	133	499
Ranking	3	1	2	4	6	7	5

**Table 3:** Respondents’ (n = 275) ranking of seven attributes for stud male selected for breeding

	Attributes of Stud Dog						
Ranking	Conformation n (%)	Health n (%)	General Temperament n (%)	Temperament for Showing n (%)	Temperament as a Companion n (%)	Longevity n (%)	Colour and Marking n (%)
1	54 (19.64)	118 (42.91)	60 (21.82)	2 (0.73)	25 (9.09)	3 (1.09)	5 (1.82)
2	55 (20.00)	70 (25.45)	63 (22.91)	7 (2.55)	38 (13.82)	16 (5.82)	2 (0.73)
3	39 (14.18)	41 (14.91)	77 (28.00)	9 (3.27)	42 (15.27)	27 (9.82)	14 (5.09)
4	46 (16.73)	11 (4.00)	26 (9.45)	33 (12.00)	58 (21.09)	50 (18.18)	20 (7.27)
5	31 (11.27)	6 (2.18)	11 (4.00)	47 (17.09)	58 (21.09)	64 (23.27)	21 (7.64)
6	12 (4.36)	1 (0.36)	8 (2.91)	82 (29.82)	15 (5.45)	60 (21.82)	61 (22.18)
7	2 (0.73)	3 (1.09)	4 (1.45)	68 (24.73)	5 (1.82)	32 (11.64)	125 (45.45)
No response	36 (13.09)	25 (9.09)	26 (9.45)	27 (9.82)	34 (12.36)	23 (8.36)	27 (9.82)
Total	275	275	275	275	275	275	275
Median	3	2	3	6	4	5	7
Mode	2	1	3	6	5	5	7
Average	2.954	1.928	2.618	5.556	3.627	4.481	5.956

**Table 4:** Respondents’ (n = 275) ranking of attributes for bitch selected for breeding.

			Attributes of Bitch					
Ranking	Conformation n (%)	Health n (%)	Temperament as a Mother n (%)	Temperament for Showing n (%)	Temperament as a Companion n (%)	Longevity n (%)	Colour and Marking n (%)	Ease of Whelping n (%)
1	49 (17.82)	130 (47.27)	19 (6.91)	2 (0.73)	51 (18.55)	2 (0.73)	4 (1.45)	8 (2.91)
2	62 (22.55)	73 (26.55)	37 (13.45)	7 (2.55)	43 (15.64)	9 (3.27)	3 (1.09)	16 (5.82)
3	38 (13.82)	26 (9.45)	59 (21.45)	9 (3.27)	39 (14.18)	21 (7.64)	10 (3.64)	43 (15.64)
4	42 (15.27)	10 (3.64)	51 (18.55)	15 (5.45)	35 (12.73)	22 (8.00)	13 (4.73)	54 (19.64)
5	21 (7.64)	3 (1.09)	45 (16.36)	28 (10.18)	31 (11.27)	39 (14.18)	12 (4.36)	58 (21.09)
6	19 (6.91)	3 (1.09)	19 (6.91)	38 (13.82)	22 (8.00)	70 (25.45)	31 (11.27)	36 (13.09)
7	7 (2.55)	2 (-.73)	10 (3.64)	72 (26.18)	15 (5.45)	52 (18.91)	57 (20.73)	20 (7.27)
8	3 (1.09)	3 (1.09)	5 (1.82)	69 (25.09)	7 (2.55)	25 (9.09)	105 (38.18)	7 (2.55)
No response	34 (12.36)	25 (9.09)	30 (10.91)	35 (12.73)	32 (11.64)	35 (12.73)	40 (14.55)	33 (12.00)
Total	275	275	275	275	275	275	275	275
Median	3	1	4	7	3	6	7	4.
Mode	2	1	3	7	1	6	8	5
Average	3.10	1.86	3.767	6.362	3.465	5.625	6.711	4.492

**Table 5:** Ordinal score ranking of 147 registered breeder’s motives for breeding of first litter.

Cluster	Breeding Motive						
	Dog Lover	Breed Specific	Dog Specific	Dog Events	Breeder Encouraged	Dog Breeding Family	Financial Gain
1 Purple	4	7	5	3	4	2	1
2 Brown	4	6.5	4	5	4	2	1
3 Green	4	7	5	5	3	2	1
4 Blue	2.5	2	4	2.5	4.5	5.5	7

**Table 6:** Level of importance placed on each aim for the 147 registered breeders (Essential = 4, Very important = 3, Somewhat important = 2, Not important = 1).

Cluster	Breeding Aim						
	Breed Standard	Healthy Companions	Breed Betterment	Original Purpose	Dog Event	Finance Breeding	Financial Gain
1 Purple	3	4	4	4	2	1	1
2 Brown	4	4	4	4	3	1	1
3 Green	4	4	4	4	3	1	1
4 Blue	2	4	4	3	2	2	1

**Table 7:** Respondents’ (n = 147) cluster medians of seven attributes for stud male selected for breeding.

Cluster	Attribute for Stud Male						
	Conformation	Health	General Temperament	Temperament for Showing	Temperament as a Companion	Longevity	Colour and Marking
1 Purple	4	6	5	2	5	3	2
2 Brown	5	6	6	2	3	4	1
3 Green	6	6	5	3	4	2	1.5
4 Blue	5	6	3	4	3.5	2.5	7

**Table 8:** Registered breeder respondents’ (n = 147) cluster medians for importance of breeding bitch attributes.

Cluster	Attributes of Breeding Bitch							
	Conformation	Health	Temperament as a Mother	Temperament for Showing	Temperament as a Companion	Longevity	Colour and Marking	Ease of Whelping
1 Purple	5	8	6	2	7	3	2	5
2 Brown	6	8	6	2	4	4	1	5
3 Green	7.5	7	5	4	5.5	2	2	3
4 Blue	4.5	1.5	2.5	7	3	4	8	5.5

**Table 9:** Respondents’ reported responsibility for the physical and mental health of puppies up until the age of three years and beyond.

Response	Health Aspect for Which Respondents Stated Taking Responsibility			
	Physical Health		Mental Health	
	<3 years n (%)	≥3 years n (%)	<3 years n (%)	≥3 years n (%)
Strongly agree	69 (25.09)	45 (16.36)	55 (20.00)	42 (15.27)
Agree	81 (29.45)	62 (22.55)	66 (24.00)	42 (15.27)
Neither agree or Disagree	59 (21.45)	76 (27.64)	70 (25.45)	81 (29.45)
Disagree	20 (7.27)	40 (14.55)	34 (12.36)	52 (18.91)
Strongly disagree	8 (2.91)	13 (4.73)	9 (3.27)	18 (6.55)
No response	38 (13.82)	39 (14.18)	41 (14.91)	40 (14.55)
Total sample	275 (100)	275 (100)	275 (100)	275 (100)

**Table 10:** Reported frequency of retention of pick female or male puppies from litters bred.

Frequency	Sex of Puppy	
	Female n (%)	Male n (%)
Always	36 (13.38)	15(5.56)
Often	110 (40.89)	48 (17.78)
Sometimes	100 (37.17)	130 (48.15)
Seldom	15 (5.58)	59 (21.85)
Never	8 (2.97)	18 (6.67)
No answer	6 (2.18)	5 (1.82)
Total sample	275	275

## Supplementary Materials

The following are available online at https://www.mdpi.com/2076-2615/10/12/2319/s1: Australian Dog Breeders’ Survey.
