# Supplementary material for: Reported Motivations and Aims of Australian Dog Breeders—A Pilot Study
Source: Animals (Basel). 2020 Dec 7;10(12):2319. doi: 10.3390/ani10122319 (PMC7762288; doi:10.3390/ani10122319)
Supplement: Supplementary file 1 [file animals-10-02319-s001.pdf]

Australian Dog Breeders Survey[1]

Introduction

Invitation
You are invited to undertake a survey which will ask you to provide answers to a number of questions primarily focusing around your breeding practices and objectives as an Australian dog breeder.

What is the purpose of this study?
The Chief Investigator is Dr Sonia Shimeld. The breeder survey is being conducted by Sonia, Professor Paul McGreevy, Professor Dianne Nicol and Simone Bingham.

An extensive literature review, case law analysis and consumer survey have identified that there are concerns around how some dogs are bred and how some dogs are sold in Australia. The consumer survey has confirmed that Australian dog owners hold strong views around how and who should be breeding dogs in Australia. To expand on the data obtained in the consumer survey and to give Australian dog breeders a voice, this breeder survey seeks to gain knowledge of the breeding objectives and motives of Australian dog breeders. It also seeks to collect data on the selling practices of dog breeders, breeders' understanding of the companion dog market and their view on the current regulation of breeding.

Why have I been invited to participate?
There are many stakeholders in dog breeding in Australia. One of the largest is dog breeders. You have been chosen as a dog breeder. Your involvement in the study is voluntary and there are no consequences if you decide not to participate.

What will I be asked to do?
You are being asked to answer up to 40 questions provided to you as an online survey through Survey Monkey. The responses you provide are confidential unless you choose to provide an email address for a follow up survey that will be conducted by the research team in 2016.

Are there any possible benefits from participation in this study?
This research aims to provide guidance on how the current regulatory framework is able to address the issues in dog breeding and how it might be changed to better address the issues.

Are there any possible risks from participation in this study?
There are no foreseeable risks of your participation in this research.

What if I change my mind during or after the study?
It is stressed that participation in this research is entirely voluntary. You may decline to answer any survey question, withdraw at any time without effect or explanation and should you so wish, also withdraw any data you supplied to date where it is identifiable, prior to submitting the survey.

What will happen to the information when this study is over?
The data from the electronic surveys will be kept secured in a password protected computer file in Simone Bingham's office at the University of Tasmania for a period of 5 years after publication after which time the data will be deleted.

What if I have questions about this study?
Should you have any questions about your participation in this study please contact either Simone Bingham (Room 325 Centenary Building, Phone 6226 2314, email Simone.Bingham@utas.edu.au), Sonia Shimeld Room (Room 309 Centenary Building, Phone 6226 7586, email Sonia.Shimeld@utas.edu.au) or Paul McGreevy (B19 – RMC Gunn Building) University of Sydney, Phone (02) 9351 3957, email paul.mcgreevy@sydney.edu.au

This study has been approved by the Tasmanian Social Sciences Human Research Ethics Committee. If you have concerns or complaints about the conduct of this study, please contact the Executive Officer of the HREC

[1] This survey was open from August to November 2015 275 responses were received

(Tasmania) Network on (03) 6226 7479 or email human.ethics@utas.edu.au. The Executive Officer is the person nominated to receive complaints from research participants. Please quote ethics reference number H0013192.

Objectives and Motivations for Breeding

1. Please rank the following statements in order of importance to indicate which **best** describes what motivated you to breed your **first litter** of puppies. (Please rank from 1 to 7, with 1 being the one that best describes what motivated you and 7 being the least relevant motivation.)
   - o My love of dogs in general
   - o My love of a specific breed of dog
   - o My love of a particular dog I wanted to breed from
   - o My love of competing in dog events
   - o Another breeder encouraged me to breed my first litter
   - o My family have been involved in dog breeding, so I continued the tradition
   - o I believed breeding would provide some financial benefit

2. How important are the following aims when you breed a litter

   Essential Very important Somewhat important Not important

   - o To breed to the Australian National Kennel Council Ltd (ANKC) Breed Standard
   - o To breed fit and healthy companion animals
   - o For the betterment of the breed in Australia
   - o To breed dogs that are fit for their original purpose (i.e., working or service dogs)
   - o To breed dogs that can win in dog events
   - o To make financial gain as a source of income
   - o To make money to continue breeding

3. What breeds/types have you bred during your time as a dog breeder in Australia? (Tick all the breeds/types you have bred) {drop down box}

4. If you were asked to rank the importance of the following attributes of stud dogs when you breed, what rank would you assign them? (Rank from 1 to 7 by clicking the most important one and so on.)
   - o Conformation
   - o Health
   - o General temperament
   - o Temperament for showing
   - o Temperament as a companion
   - o Longevity
   - o Colour and Markings

5. If you were asked to rank the importance of the following attributes of bitches when you breed, what rank would you assign them? (Rank from 1 to 8 by dragging the most important to the top and clicking one and so on.)
   - o Conformation
   - o Health
   - o Temperament as a mother
   - o Temperament for showing
   - o Temperament as a companion
   - o Ease of whelping
   - o Longevity
   - o Colour and Markings

6. When you breed, how often do you retain a **female** pick of the litter?

   Always Often Sometimes Seldom Never

7. When you breed, how often do you retain a **male** pick of the litter?

Always Often Sometimes Seldom Never

Questions about Puppy Buyers

8. How do you believe purchasers of a companion puppy **rank** the importance of the following **behavioural** attributes in their **preferred breed**? (Rank from 1 to 7 by clicking the most important and inserting one and so on)
    - Obedient
    - Friendly
    - Affectionate
    - Healthy
    - Loyal
    - Good with children
    - Good with other animals

9. How do you believe purchasers of a companion puppy rank the importance of the following **physical** attributes of **pups** you present for sale? (Rank from 1 to 4 by dragging the most important to the top and clicking one and so on)
    - Sex
    - Colour and Markings
    - Conformation
    - Health

10. How do you believe purchasers of a companion puppy rank the importance of the following **behavioural** attributes of **pups** you present for sale? (Rank from 1 to 4 by dragging the most important to the top and clicking one and so on)
    - Boldness
    - Calmness
    - Playfulness
    - Friendliness

11. Approximately what percentage of prospective puppy purchasers want the pick of the litter?

    100 – 75% 74 – 50% 49 – 25% 24 – 1%

12. Approximately what percentage of prospective puppy purchasers are prepared to take the last pup of the litter?

    100 – 75% 74 – 50% 49 – 25% 24 – 1%

13. Approximately what percentage of prospective puppy purchasers want the boldest pup in the litter?

    100 – 75% 74 – 50% 49 – 25% 24 – 1%

Questions about Selling Puppies – general

14. How did you advertise your last litter? (Tick as many as apply)

    - Breed Club Newsletter and other Club resources
    - Canine Association breeder directories
    - DogzOnline.com.au

- Gumtree.com.au
- Printed newspaper
- Social Media
- Through a commercial wholesaler or pet shop approved by the Pet Industry Association
- Other online breeder listing pages
- Own web page
- Word of Mouth
- Other (please specify)

15. For your last litter, did you pay any of the following (Tick as many as apply)
    - Stud fee
    - Lease fee for the bitch
    - Transport cost for the bitch for the mating
    - Transport cost for dog for the mating
    - Veterinarian fees for the mating or pregnancy (such as for an ultrasound)
    - Veterinarian fees associated with the birth
    - Microchipping fees for the puppies
    - Vaccination fees for the puppies
    - Worming product for the puppies
    - Registration fees for the litter and for each puppy
    - Expenses to create a “puppy pack” for each new owner
    - Transport costs for puppies (not otherwise paid by the puppy buyer)
    - Other (please specify or provide more details of any other expenses you incurred for your last litter)

16. What do you currently provide when you sell your puppies?
    - o Yes
    - o No

Questions about Selling Puppies – key terms

17. What are some of the key terms/items you include (tick as many as you include)
    - o Conformation of price
    - o Confirmation of puppy’s pedigree and registration details
    - o Confirmation of health testing
    - o Confirmation of vaccination
    - o Microchip details
    - o Return-puppy clause should the buyer be unable to keep the puppy
    - o Details on how the puppy has been raised
    - o Information around the puppy's behavioural and / or physical attributes
    - o Guarantees around the puppy's health

18. Would you support the introduction of mandatory puppy contracts? These could include such things as - the information you provide to potential puppy buyers upon which they make their buying decisions, confirmation of the heath tests that you undertake, information around how the puppy was raised and what vaccination he or she has had prior to leaving your premises. They could also contain a clause that encourages new owners to return the puppy should they not be able to keep it.

    - o Yes
    - o No

19. Which best confirms why you do not support the introduction of puppy contracts?
    - Too costly

- Too time-intensive
- I believe the way I sell my puppies currently works well, so a puppy contract is not required
- A puppy contract would not be effective
- I do not believe a puppy buyer should have the protection of a puppy contract

None of these set out the reason I do not support the introduction of a puppy contract.
(If this is your answer, please provide details here of the reason)

20. Do you have a return policy should an owner not wish to keep a puppy or dog bred by you?
    - Yes
    - No

21. Which answer best confirms why you do not have a return policy?
    - Once a puppy has left me I have no way of knowing how it has been cared for or treated
    - There are a number of options available for puppy buyers who do not wish to retain a puppy, including advertising to find a new home
    - I do not have the room or capacity to have puppies coming back to me
    - I do offer assistance and recommendations for where the buyer can find a new home, so do not need to take the puppy back
    - It would be too expensive for me to have this policy in place for all puppies I breed
    - Other (please specify)

22. What do you currently provide when you sell your puppies? (Tick as many as apply)
    - Written receipt for funds paid
    - Written or emailed information on the history of the breed
    - Written or emailed information about health, lifestyle and dietary requirements
    - Pedigree / Registration document
    - Vaccination certificate
    - Microchip certificate / change over document
    - Deworming tablets
    - Blanket
    - Crate
    - Toy
    - Food
    - Other (please specify)

23. We are interested in what you think is your role in the health of puppies you have sold. Please consider the following statements and provide your level of agreement or disagreement with them.

- I take responsibility for the short-term physical health (up to 3 years of age) of the dogs I have sold

  Strongly agree   Agree   Neither agree nor disagree   Disagree   Strongly disagree

- I take responsibility for the long-term physical health (beyond 3 years of age) of the dogs I have sold

  Strongly agree   Agree   Neither agree nor disagree   Disagree   Strongly disagree

- I take responsibility for the short-term mental health (up to 3 years of age) of the dogs I have sold

  Strongly agree   Agree   Neither agree nor disagree   Disagree   Strongly disagree

- I take responsibility for the long-term mental health (beyond 3 years of age) of the dogs I have sold

  Strongly agree   Agree   Neither agree nor disagree   Disagree   Strongly disagree

24. What health testing do you undertake in prospective breeding stock? (Tick as many as apply)
   - Elbow and hip dysplasia
   - Progressive Retinal Atrophy
   - Collie Eye Anomaly
   - Cataracts
   - Legge Perthe's disease
   - None
   - Other (please specify)

The Economics of Breeding

25. In 2014, how many litters did you breed? [Dropdown number to tick]

26. In 2014 what was the total number of live puppies born in all the litters you had? [Dropdown number to tick], 1 – 100 and More than 100

27. Considering the litter with the **largest number of live puppies** that you bred in 2014:
   - o What breed was this litter?
   - o How many puppies were born in this litter?
   - o How many live puppies were in the litter?
   - o How many puppies did you sell?
   - o How many of the puppies that you sold went to companion homes?
   - o How many were sold to dog-event homes?
   - o How many were sold as working or service dogs?
   - o What price did you sell these puppies for?
   - o Did any of these puppies come back to you for any reason?
   - o How many pups from this litter do you retain today?

   If you would like to provide more details, please do so here: ………………………………………………

28. When you breed, do you keep accurate records of expenses incurred?
   - o Yes
   - o No
   - o

29. If yes, would you be prepared to be involved in a further study we are conducting in an attempt to quantify the economic benefits and costs associated with breeding?
   - o Yes
   - o No

30. If you are prepared to be involved in a further research project please cut and paste the following link into a new url and you can leave your contact details there.
   https://www.surveymonkey.com/r/economicsofbreeding

Economics of Breeding - Practices

31. How many bitches that you have bred from in the past 5 years still reside with you?
   - o All of them
   - o Most of them
   - o Approximately half of them
   - o Fewer than half
   - o None
   - o Other (please specify)

32. How many brood bitches under 6 years of age do you currently own? {Drop down to 20 and then Over 20}

33. How often do you believe a bitch of the breed that had your largest litter in 2014 should be bred from over her lifetime? {Drop down list to 10}

34. Have you ever declared any money earned by you from breeding and selling puppies as income at the Australian Taxation Office?
   - Yes
   - No
   - If you would like to expand please do so

35. Is your breeding recognised as a business by the Australian Taxation office?
   - Yes
   - No

36. Which of the following best describes your breeding?
   - A hobby
   - A small business
   - A commercial breeding enterprise
   - Other (please specify)

Regulation of Dog Breeding

37. Are you aware of your rights and obligations as a dog breeder?
   - Fully aware
   - Somewhat aware
   - Not very aware
   - Not at all aware
   - Other (please specify)

38. Do you believe the current regulation of dog breeding in Australia is effective?
   - Yes
   - No
   - If you would like to expand on your answer please do so here

Membership of a Breeding Association / industry Organisation and Demographics

39. Are you currently a financial member of any of the State or Territory Canine Associations?
   - Yes
   - No

40. If you answered Yes, which ones (Please tick as many as apply)
   - Dogs ACT
   - Dogs West
   - Dogs Queensland
   - Dogs NT
   - Dogs NSW
   - Dogs SA
   - Dogs Tasmania
   - Dogs Victoria

41. Approximately how long have you been a member? [Dropdown list years <1 to 50>]

42. Your age
   - 17 or younger

- 18-20
- 21-29
- 30-39
- 40-49
- 50-59
- 60 or older

43. State [Dropdown list]

Thank you very much for completing this survey.

Submission of a completed survey form implies consent to participate in this study.

Should you wish to receive more information about the survey please email Simone on Simone.Bingham@utas.edu.au
